# Nicorandil attenuates carotid intimal hyperplasia after balloon catheter injury in diabetic rats

**DOI:** 10.1186/s12933-016-0377-6

**Published:** 2016-04-08

**Authors:** Ying Qian Zhang, Feng Tian, Ying Zhou, Yun Dai Chen, Bo Li, Qiang Ma, Ying Zhang

**Affiliations:** Department of Cardiology, Chinese PLA General Hospital, 28 Fuxing Rd, Beijing, 100853 China

**Keywords:** Nicorandil, Intimal hyperplasia, Diabetes mellitus, ATP-sensitive potassium channel, Protein kinase C

## Abstract

**Background:**

Diabetic patients suffer from undesired intimal hyperplasia after angioplasty. Nicorandil has a trend to reduce the rate of target lesion revascularization. However, whether nicorandil inhibits intimal hyperplasia and the possible mechanisms underlying it remain to be determined. We aimed at assessing the effect of nicorandil on intimal hyperplasia in diabetic rats.

**Methods:**

After intraperitoneal injection of streptozotocin (STZ, 50 mg/kg), balloon injury model was established in carotid arteries of diabetic rats. Rats were randomized to vehicle, nicorandil (15 mg/kg/day) or 5-hydroxydecanoate (5-HD, 10 mg/kg/day), a mitochondrial ATP-sensitive potassium channel (mitoK_ATP_ channel)-selective antagonist. Perivascular delivery of εPKC siRNA was conducted to determine the role of εPKC pathway in intimal hyperplasia. In hyperglycemia environment (25 mM glucose), primary culture of vascular smooth muscle cells (VSMCs) were treated with nicorandil or 5-HD. Cell proliferation and cell migration were analyzed.

**Results:**

Intimal hyperplasia significantly increased 14 days after balloon injury in diabetic rats (p < 0.01). Nicorandil inhibited intima development, reduced inflammation and prevented cell proliferation in balloon-injured arteries (p < 0.01). The protective effects of nicorandil were reversed by 5-HD (p < 0.05). εPKC was activated in balloon-injured arteries (p < 0.01). Nicorandil inhibited εPKC activation by opening mitoK_ATP_ channel. Perivascular delivery of εPKC siRNA inhibited intimal hyperplasia, inflammation and cell proliferation (p < 0.01). High glucose-induced VSMCs proliferation and migration were inhibited by nicorandil. εPKC activation induced by high glucose was also inhibited by nicorandil and that is partially reversed by 5-HD. εPKC knockdown prevented VSMCs proliferation and migration (p < 0.01).

**Conclusions:**

Our study demonstrates that nicorandil inhibits intimal hyperplasia in balloon-injured arteries in diabetic rats. Nicorandil also prevents VSMCs proliferation and migration induced by high glucose. The beneficial effect of nicorandil is conducted via opening mitoK_ATP_ channel and inhibiting εPKC activation.

**Electronic supplementary material:**

The online version of this article (doi:10.1186/s12933-016-0377-6) contains supplementary material, which is available to authorized users.

## Background

Intimal hyperplasia occurs after percutaneous coronary intervention (PCI) or percutaneous transluminal coronary angioplasty (PTCA). It is the pathologic basis for restenosis. Diabetes mellitus (DM) promotes intimal hyperplasia via increased inflammation, proliferation, and oxidative stress in lesions [1] and results in higher risk of restenosis [2]. Nicorandil is a hybrid agent with ATP-sensitive potassium channel (K_ATP_) channel opener and nitrate properties. Retrospective trials reveal that in patients underwent PCI or PTCA, nicorandil reduces the rate of target vessel revascularization (TVR) [3]. In diabetic patients, a trend toward lower target lesion revascularization (TLR) was observed in nicorandil treated group [4]. Besides, nicorandil has been found to stabilize coronary plaque [5] and reduce coronary artery disease mortality [6] in patients with stable angina.

Intimal hyperplasia is characterized by vascular smooth muscle cell (VSMC) proliferation, inflammatory cell infiltration, endothelial cell injury and augmented position of extracellular matrix. εPKC regulates VSMC proliferation and migration in vitro and is involved in the development of intimal hyperplasia in vivo [7]. A recent study finds that hyperpolarization of mitochondrial membrane potential (ΔΨm) promotes VSMCs proliferation and intimal hyperplasia [8] in several preclinical animal models. Nicorandil is K_ATP_ channel opener. It directly opens the mitochondrial K_ATP_ channel (mitoK_ATP_ channel) and depolarizes ΔΨm [9, 10]. Chronic hyperglycemia leads to an increase in PKC activation that could promote VSMCs growth and mediates restenosis [1]. In rat myocardial infarction model, nicorandil inhibits εPKC activation by opening the mitoK_ATP_ channel [11]. Nicorandil has also been found to inhibit oxidative stress [12] and attenuate inflammation [13]. However, whether nicorandil inhibits intimal hyperplasia after balloon catheter injury in diabetic rats remains to be determined.

In the present study, we hypothesizes that nicorandil inhibits intimal hyperplasia in diabetic rats through opening mitoK_ATP_ channel and inhibiting εPKC activation. An in vivo carotid balloon catheter injury model and in vitro primary cultured VSMCs were used to investigate this hypothesis.

## Methods

### Induction of diabetes and rat balloon catheter injury model

The animal experiments were approved by Animal Research Committee of Chinese PLA General Hospital. All experiments were conducted in accordance with the Guide for the Care and Use of Laboratory Animals published by the US National Institutes of Health (NIH Publication No.85-23, revised 1996). Male Sprague-Dawley (SD) rats (n = 60, 150–180 g) were purchased from Experimental Animal Center of Chinese PLA General Hospital (approval No. SCXK 20120001). All animals were housed in a 12 h light/dark cycle room at controlled temperature (23 ± 2 °C) and humidity (50–60 %) with a free access to food and water. All rats were fed with regular rodent chow for the first week. After 2 weeks of high fat diet (85 % standard diet, 8 % animal fat, 2 % total cholesterol, 5 % glucose), DM was induced by a single intraperitoneal injection of citrate buffer vehicle (0.1 M, pH 4.5) with streptozotocin (50 mg/kg; Sigma-Aldrich, St Louis, MO, USA). The diabetic model was confirmed after 72 h by random blood glucose >16.7 mmol/L [1]. The balloon catheter injury model was created with a 2F Fogarty catheter (Edwards Lifesciences, Irvine, CA) in the left common carotid artery as previously described [14]. Briefly, rats were anesthetized by intraperitoneal injection of pentobarbital (50 mg/kg, Sigma-Aldrich, St Louis, MO, USA). The left external carotid artery was exposed. Then the balloon was introduced through the left external carotid artery into the common carotid artery. The common carotid artery was denudated by passing the inflated balloon through the lumen three times. After removal of the catheter, the punched area was sealed, and the common carotid artery resumed blood flow.

Sham group (n = 8) was conducted with an uninflated balloon and treated by gavage feeding with vehicle. Balloon injury group (n = 10) was treated by gavage feeding with vehicle from the 1st day after balloon injury. Nicorandil group (n = 10) was treated by gavage feeding with nicorandil (15 mg/kg/day, Chugai Pharmaceutical Co., Japan) from the 1st day after balloon injury. 5-hydroxydecanoate (5-HD; Sigma-Aldrich, St Louis, MO, USA), a mitoK_ATP_ channel-selective antagonist was used to detect the role of mitoK_ATP_ channel. Nicorandil and 5-HD group (n = 10) was treated by gavage feeding with nicorandil and 5-HD (10 mg/kg/day) from the 1st day after balloon injury. 14 days after balloon injury, rats were killed with excessive anesthesia and the left common carotid arteries were subjected to following studies.

### Histology

Carotid arteries were fixed in 4 % paraformaldehyde, dehydrated and embedded in paraffin. 5-μm thick sections were prepared. Serial three cross sections were cut from each artery and stained with Elastica van Gieson to observe the elastic laminae and intimal hyperplasia. Intima, media and adventitia cross-sectional areas were measured by software Image J 1.49. The intima area was calculated by subtracting the lumen area from the area defined by the internal elastic lamina (IEL). The media area were calculated by subtracting the area defined by the IEL from the area defined by the external elastic lamina (EEL) [15]. VSMCs proliferation was detected by immunofluorescent (IF) staining of proliferating cell nuclear antigen (PCNA; Cell Signaling Technology, MA, USA, 1:200) and alpha smooth muscle actin (α-SMA, Abcam, Cambridge, UK, 1:200). Inflammation was detected by immunohistochemistry (IHC) staining of CD68 (Abcam, Cambridge, UK, 1:200).

### In vitro VSMCs culture and high glucose treatment

VSMCs was isolated from rat aorta and cultured in DMEM containing 5.5 mM glucose supplemented with 10 % FBS at 37 °C under 5 % CO_2_ in a humidified incubator. VSMCs were identified by staining with α-SMA. The 3rd to 5th passages were used for experiments. To mimic the hyperglycemia environment, VSMCs cultured in 5.5 mM glucose were serum starved for 24 h and then incubated in 25 mM glucose culture medium [1]. To determine the effect of nicorandil and mitoK_ATP_ channel on VSMCs, nicorandil (100 μM, Sigma-Aldrich, St Louis, MO, USA) and 5-HD (500 μM, Sigma-Aldrich, St Louis, MO, USA) were used in cell culture. In control group, VSMCs were incubated in 5.5 mM glucose culture medium containing 1 % FBS for 24 h. In high glucose group, VSMCs were incubated in 25 mM glucose culture medium containing 1 % FBS for 24 h. In high glucose-nicorandil group, VSMCs were incubated in 25 mM glucose culture medium containing 100 μM nicorandil and 1 % FBS for 24 h. In high glucose-nicorandil-5HD co-treatment group, 500 μM 5-HD diluted in 25 mM glucose culture medium was given 30 min earlier than nicorandil.

### Small interfering RNA transfection in vitro and in vivo

Perivascular delivery of εPKC siRNA was conducted as previously described [16, 17]. In-vivo ready εPKC siRNA (NM_017171.1), (#AM16830, ThermoFisher, Wilmington, DE) were premixed with lipofectamine rnaimax (Life Technologies, NY) and optimem (Invitrogen, Carlsbad, CA) to a total volume of 60 μl, and then mixed with 80 μl 4 % pluronic gel (Sigma-Aldrich, St Louis, MO, USA). Following dissecting the carotid artery from the connecting tissue and conducting the balloon injury, 140 μl ice-cold mixed pluronic gel containing either scramble siRNA (100 μg) or εPKC siRNA (100 μg) applied to the carotid artery. The incision was sutured after the application of the gel. The scramble siRNA group (n = 10) and εPKC siRNA group (n = 10) were removed 14 days after the balloon injury. For the transfection of εPKC siRNA (NM_017171.1), (#AM16708, ThermoFisher, Wilmington, DE) and non-targeted siRNA in vitro, VSMCs at 60 % confluence were transfected at the concentration of 25 nM using lipofectamine rnaimax (Life Technologies, NY) according to the manufacture’s instruction. 48 h after siRNA transfection, CMECs were harvested for further experiments.

### Cell proliferation and migration

Cell proliferation was determined by a modified 3-(4,5-dimethyl-thiazol-2-yl)-2,5-dyphenyltertrazolium bromide (MTT) assay (Sigma-Aldrich, St Louis, MO, USA) and BrdU Cell Proliferation Assay Kit (Cell Signaling Technology, MA, USA). VSMC migration was analyzed by wound healing assay. VSMCs (50–70 % confluence) were wounded uniformly by using 1.15 mm diameter pipette tip. 24 h later, standard photographs of the wound areas were taken by a phase contrast microscopy (Olympus Corporation, Tokyo, Japan). Distance between cells of the wound was calculated by subtracting the distance at the lesion edge at 24 h from that at 0 h [18].

εPKC translocation in vivo and in vitro was assessed by western blot. Carotid arteries were frozen in liquid nitrogen and stored at −80 °C until western blot. In brief, arteries were homogenized in cold buffer (50 mM Tris-HCl (pH 7.4), 5 mM EDTA, 10 mM EGTA, 50 mM NaF, 50 μg/ml phenylmethylsulfonyl fluoride, and 0.3 % β-mercaptoethanol). The supernatant centrifugated at 1000*g* for 10 min was recentrifuged at 100,000*g* for 60 min at 4 °C. The 100,000*g* supernatant was the cytosolic fraction. Particulate fractions were obtained by treating the 100,000*g* pellet with 3 % Triton X-100 and recentrifugation at 10,000*g* for 10 min [19]. In VSMCs, cells were collected, homogenized in homogenization buffer (20 mM Tris-HCl (pH 7.4), 2 mM EDTA,10 mM EGTA, 250 mM sucrose, 1× phosphatase inhibitor cocktail (Cell Signaling Technology, MA, USA). Cell homogenates were centrifuged at 100,000*g* for 30 min and supernatants were collected as soluble samples. The pellets were homogenized with homogenization buffer containing 1 % Triton X-100 and recentrifugation at 10,000*g* for 30 min. The supernatants are particulate fractions which is activated εPKC [7]. Cytosolic and particulate fractions were separated by SDS-PAGE, transferred to polyvinylidene difluoride membranes, and probed with antibodies for εPKC (Santa Cruz, CA, USA). The primary antibodies at the concentration of 1:1000 were exposed for overnight at 4 °C. Next, horseradish peroxidase-conjugated secondary antibodies (Beyotime, China) at the concentration of 1:5000 were added, and incubated for 1 h at 37 °C. The membranes were then developed by enhanced chemiluminescence (Beyotime, China). The same membranes were reprobed with antibody for actin (Beyotime, China). The blotting film was quantified using a scanner and a densitometry program (Image J).

### Statistical analysis

Data were presented as mean ± SE. The statistic software package SPSS 13.0 was used for analysis of data. Statistical comparisons were performed using the paired, two-tailed Student’s t test for experiments consisting of two groups only. One-way ANOVA with post hoc testing were used for experiments consisting of more than two groups. If normality test failed, Kruskal–Wallis with Dunn’s post hoc test was used. Results were considered statistically significant when p < 0.05.

## Results

### Carotid balloon injury is established in DM rats

**Fig. 1:**
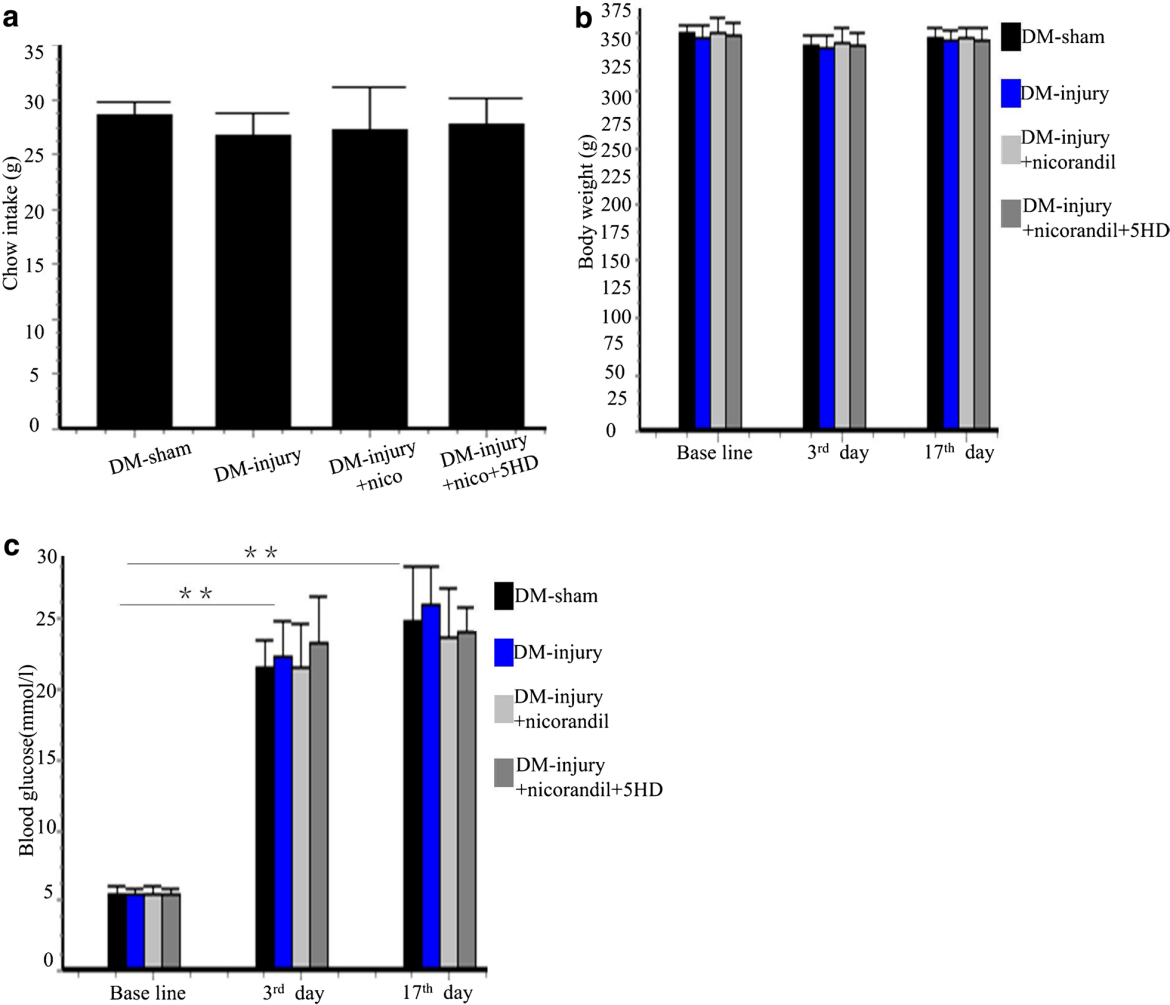
Chow intake, body weight and blood glucose in each group. **a** Chow intakes in different groups. No significant difference was observed among different groups. *Bars* represent mean ± SE. **b** Body weight in sham operation group (DM-sham group, n = 8), balloon injury group (DM-injury group, n = 10), nicorandil-treated balloon injury group (DM-injury + nicorandil group, n = 10), and nicorandil and 5-HD-treated group (DM-injury + nicorandil + 5HD group, n = 10). **c** Blood glucose in DM-sham group, DM-injury group, DM-injury + nicorandil group, and DM-injury + nicorandil + 5HD group. No significant difference was observed among different groups. Blood glucose significantly increased after STZ injection. *Bars* represent mean ± SE. **p < 0.01

Two rats with random blood glucose were excluded 3 days after STZ injection. The balloon injury procedure was performed at the 3rd day after STZ injection and was well tolerated by the diabetic rats. All animals survived the study period. There were no significant differences between chow intakes of different groups (Fig. 1a). Body weight and blood glucose were measured before STZ injection, at the 3rd day and 17th day after STZ injection, respectively. Blood glucose levels in STZ-injection rats increased 3 days after STZ injection and remained higher than 16.7 mmol/L. Nicorandil had no significant influence on body weight or glucose levels (p > 0.05) (Fig. 1b, c).

### Nicorandil attenuates intimal hyperplasia

**Fig. 2:**
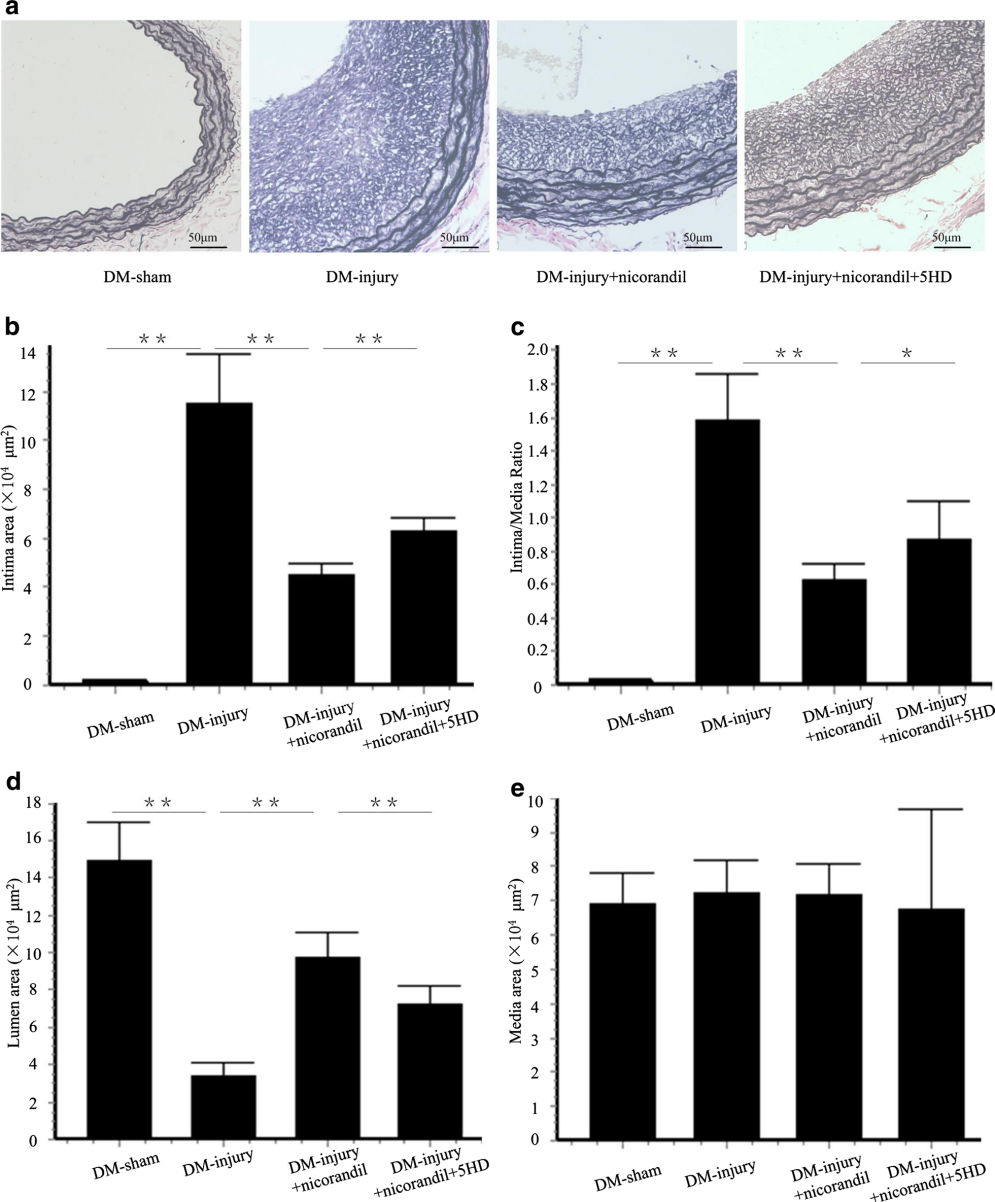
Intimal hyperplasia 14 days after balloon injury. **a** Cross sections of carotid arteries from diabetic rats 14 days after balloon injury. Sections were stained with Elastica van Gieson. **b** Quantitative analysis of intima area. **c** Quantitative analysis of intima/media area ratio. **d** Quantitative analysis of lumen area. **e** Quantitative analysis of media area in DM-sham group, DM-injury group, DM-injury + nicorandil group, and DM-injury + nicorandil + 5HD group. Nicorandil reduced the intima area and intima/media ratio, and it increased lumen area. The protective effect of nicorandil was significantly blocked by 5-HD. No significant difference was seen in media areas of different groups. *Bars* represent mean ± SE. **p < 0.01, *p < 0.05

As reported earlier, intimal hyperplasia developed in carotid arteries 14 days after balloon injury [17, 20]. A significant increase of intimal hyperplasia was observed in the DM-injury group (intima/media (I/M) ratio 1.59 ± 0.28; intimal area 11.49 ± 2.05 × 10^4^ μm^2^; lumen area 3.37 ± 0.72 × 10^4^ μm^2^) compared with the DM-sham group (I/M ratio 0.02 ± 0.01; intimal area 0.12 ± 0.05 × 10^4^ μm^2^; lumen area 14.91 ± 2.01 × 10^4^ μm^2^, p < 0.01). Nicorandil significantly reduced intimal hyperplasia (I/M ratio 0.62 ± 0.10; intimal area 4.52 ± 0.48 × 10^4^ μm^2^; lumen area 9.78 ± 1.35 × 10^4^ μm^2^, p < 0.01) compared with the DM-injury group. 5-HD, the mitoK_ATP_ channel-selective antagonist, induced more prominent intimal proliferation (I/M ratio 0.88 ± 0.22; intimal area 6.27 ± 0.53 × 10^4^ μm^2^; lumen area 7.27 ± 0.97 × 10^4^ μm^2^, p < 0.05) than that in the DM-injury + nicorandil group (Fig. 2a–d). There were no significant differences between media areas of different groups (Fig. 2e).

### Nicorandil alleviates inflammation and proliferation

**Fig. 3:**
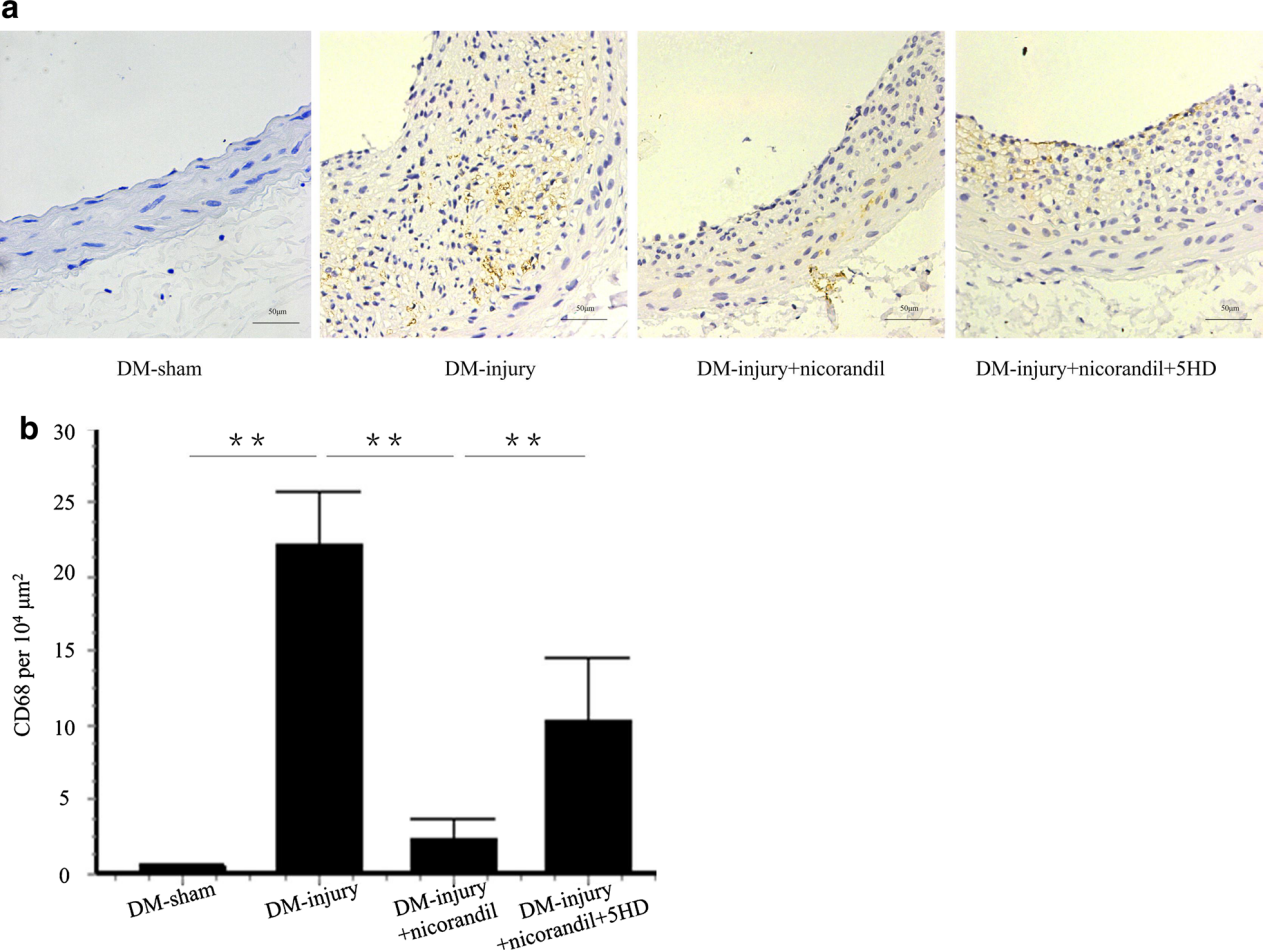
Inflammatory cell infiltration in injured carotid arteries. **a** IHC staining of CD 68 in DM-sham group, DM-injury group, DM-injury + nicorandil group, and DM-injury + nicorandil + 5HD group. **b** Quantitative analysis of CD 68-posotive cells per 10^4^ μm^2^. Nicorandil decreased CD 68-positive macrophages in balloon injured arteries. This decrease was reversed by 5-HD. *Bars* represent mean ± SE. **p < 0.01

**Fig. 4:**
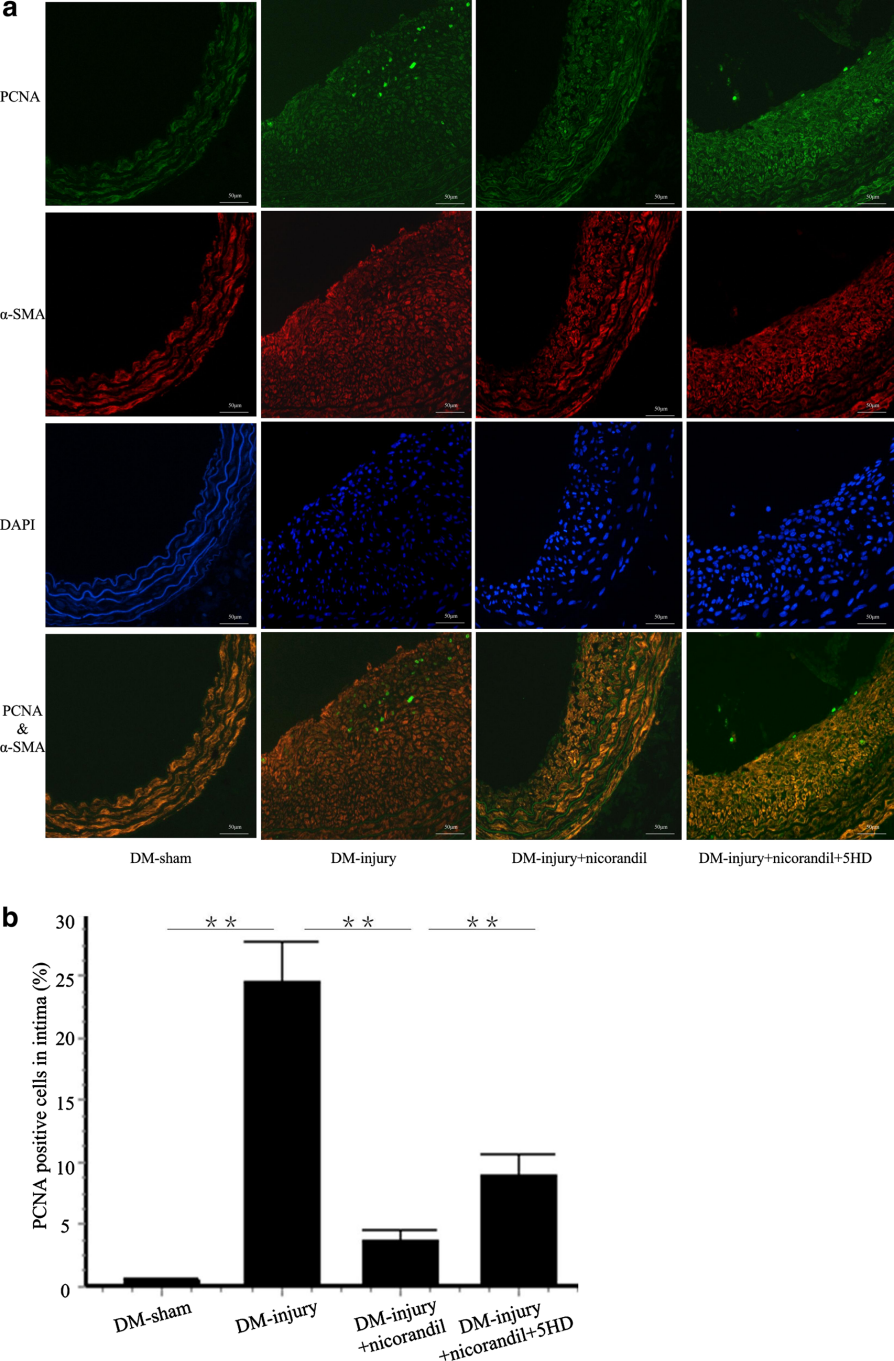
Cell proliferation in injured carotid arteries. **a** IF staining of PCNA and α-SMA in DM-sham group, DM-injury group, DM-injury + nicorandil group, and DM-injury + nicorandil + 5HD group. **b** Quantitative analysis of percentage of PCNA-positive cells in intima. Nicorandil decreased cell proliferation in balloon injured arteries. This decrease was reversed by 5-HD. *Bars* represent mean ± SE. **p < 0.01

Inflammatory cell infiltration is a key element in intimal hyperplasia after vascular injury [21]. Inflammation was assessed by IHC staining of CD68, a specific surface marker of macrophage. We observed a dramatic decrease in the number of CD68-positive cells in response to nicorandil treatment (2.42 ± 1.32 vs. 22.23 ± 3.63, p < 0.01). Additional treatment of 5-HD (10.34 ± 4.23, p < 0.01) significantly increased macrophage infiltration in intima compared with the DM-injury + nicorandil group (Fig. 3a, b). The rate of PCNA-positive cells was significantly lower in the DM-injury + nicorandil group (3.71 ± 0.85 %) than that in DM-injury group (24.6 ± 3.23 %, p < 0.01). The proliferation VSMCs in intima were evaluated by PCNA and α-SMA staining. VSMCs account for 93.6 ± 1.72 % of the ultimate intimal proliferation. The decrease of PCNA-positive cells induced by nicorandil was partially reversed by 5-HD pretreatment (9.03 ± 1.67 %, p < 0.01) (Fig. 4a, b).

### Nicorandil inhibits VSMCs proliferation and migration induced by high glucose

**Fig. 5:**
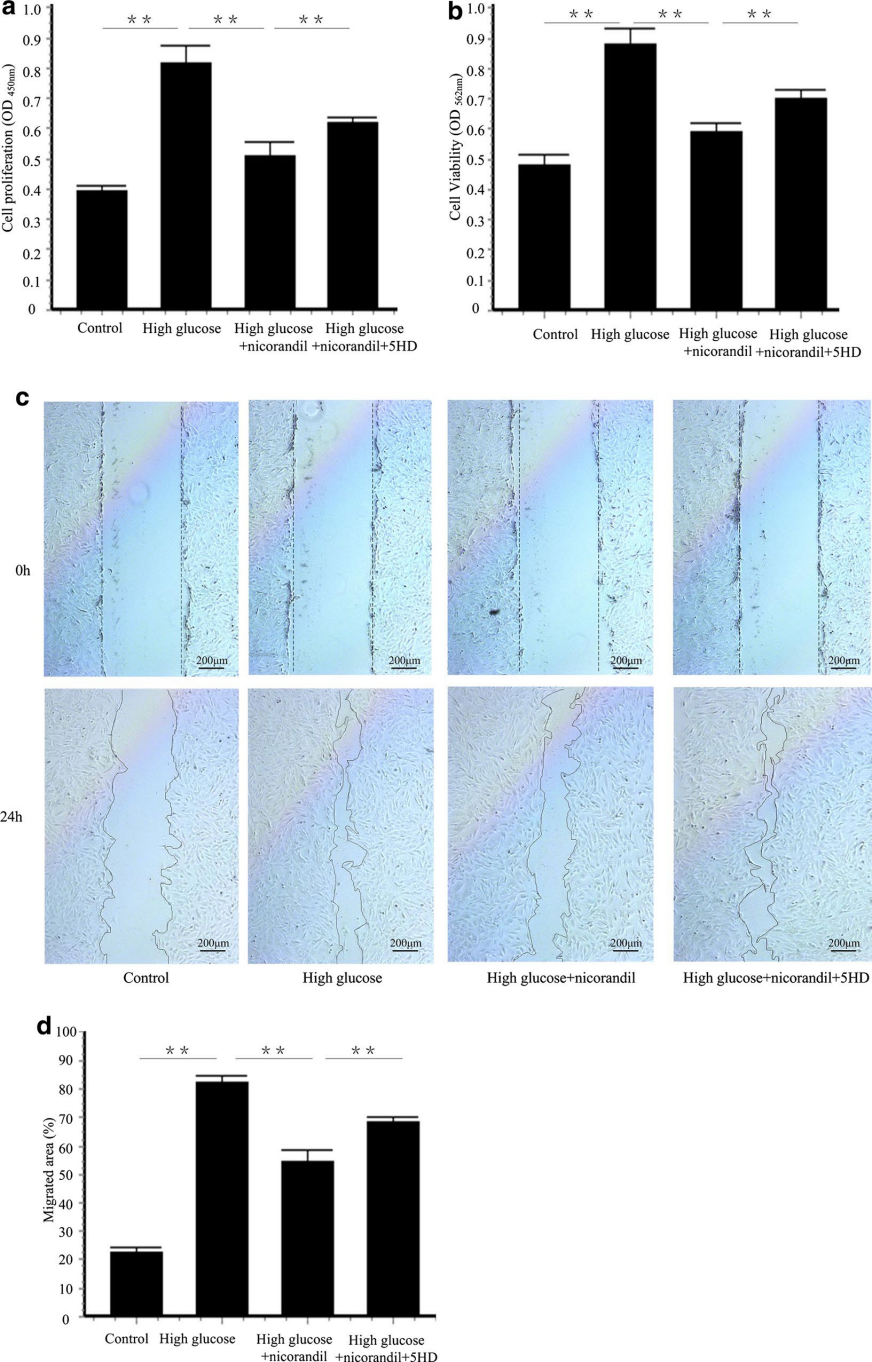
Effect of nicorandil on high-glucose-induced VSMCS proliferation and migration. **a** VSMCs were incubated with high glucose (25 mM) and treated with nicorandil (100 μmol/l) or 5-HD (500 μmol/l). VSMCs proliferation was detected using BrdU proliferation assay kit. 24 h of high glucose induced VSMCs proliferation. Nicorandil inhibited the proliferation and 5-HD partially blocked the effect of nicorandil. **b** Cell viability was assessed by MTT assay kit. Nicorandil suppressed the increase of cell viability induced by high glucose. 5-HD, a mitoKATP channel-selective antagonist, blocked the effect of nicorandil. **c** VSMCs migration was assessed by wound healing assay. **d** Quantitative analysis of percentage of migrated area in control group, high glucose group, high glucose + nicorandil group, and high glucose + nicorandil + 5-HD group. Nicorandil prevented high glucose-induced cell migration by opening mitoKATP channel. *Bars* represent mean ± SE. **p < 0.01

To investigate the mechanisms of therapeutic potential of nicorandil on intimal hyperplasia after balloon injury in diabetic models, VSMCs isolated from thoracic aorta were used. Cell proliferation was demonstrated by BrdU and MTT assay. Nicorandil prevented cell proliferation induced by high glucose. Pretreatment of 5-HD alleviated the decrease of cell proliferation induced by nicorandil (Fig. 5a, b). These results demonstrated that nicorandil suppressed VSMCs proliferation and cell viability by opening mitoK_ATP_ channel. VSMCs migration also contributes to intima hyperplasia after arterial injury [22]. A wound healing assay was performed in VSMCs stimulated with high glucose. Cell migrated more slowly in the present of nicorandil 24 h post high glucose stimulation (54.50 ± 3.91 vs. 82.32 ± 2.33 %, p < 0.01). 5-HD increased the migrated area (68.35 ± 2.04 %, p < 0.01) compared with the nicorandil treated group (Fig. 5c, d).

### εPKC is activated in intimal hyperplasia in balloon-injured arteries

**Fig. 6:**
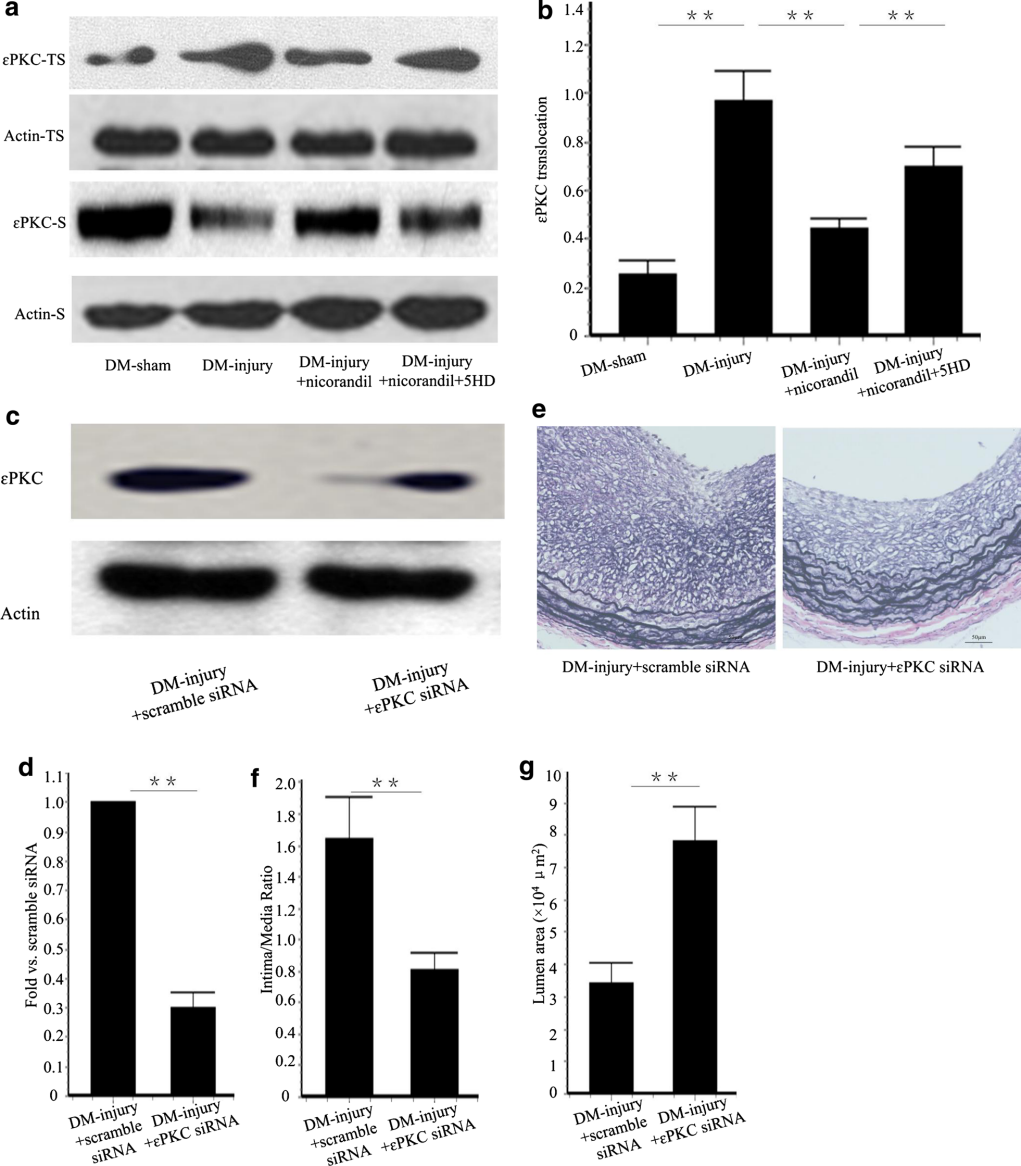
Effect of εPKC on intimal hyperplasia in balloon-injured carotid arteries. **a** Representative western blots for εPKC translocation in injured carotid artery treated with or without nicorandil or 5-HD. **b** Quantitative analysis of εPKC translocation. εPKC translocation from the cell soluble (S) to the cell particulate fraction (TS) was observed after balloon injury. This translocation was blocked by nicorandil. The effect of nicorandil was reversed by 5-HD. **c** Representative western blots for εPKC protein in the injured carotid artery with or without εPKC siRNA. **d** Quantitative analysis of εPKC protein. εPKC expression in εPKC siRNA delivered group was reduced to 30.41 ± 5.17 % of that in the scramble siRNA delivered group e: Representative cross-section of carotid artery stained with Elastica van Gieson. **f** Quantitative analysis of of intima/media ratio. **g** Quantitative analysis of lumen area in DM-injury + scramble siRNA group and DM-injury + εPKC siRNA group. Knockdown of εPKC reduced the intima/media ratio and increased lumen area. *Bars* represent mean ± SE. **p < 0.01

It has been reported that chronic hyperglycemia leads to PKC activation [1]. Recent study reveals that sustained inhibition of εPKC inhibits intimal hyperplasia in rat models [7]. Thus, we investigated whether nicorandil inhibited εPKC activation in injured arteries in diabetic rats. εPKC translocation (movement from cell soluble to cell particulate fraction) is an established method to assess εPKC activation. We found that carotid balloon injury in diabetic rats stimulated εPKC activation, and this activation was partly blocked by gavage feeding with nicorandil. Furthermore, 5-HD partly reversed the blockage exhibited by nicorandil (Fig. 6a, b). εPKC was knockdown by perivascular delivery of εPKC siRNA, and the silencing efficiency was determined by western blot. εPKC expression in εPKC siRNA delivered group was reduced to 30.41 ± 5.17 % of that in the scramble siRNA delivered group (Fig. 6c, d). Intimal hyperplasia was significantly inhibited when εPKC siRNA was perivascular delivered immediately after carotid injury. Lumen area increases from 3.41 ± 0.62 × 10^4^ μm^2^ to 7.80 ± 1.08 × 10^4^ μm^2^ (p < 0.01) after εPKC knockdown. I/M ratio was lower in the εPKC siRNA delivered group (0.82 ± 0.10) than that in the scramble siRNA delivered group (1.62 ± 0.54, p < 0.01) (Fig. 6e–g).

### εPKC activation results in inflammation and proliferation

**Fig. 7:**
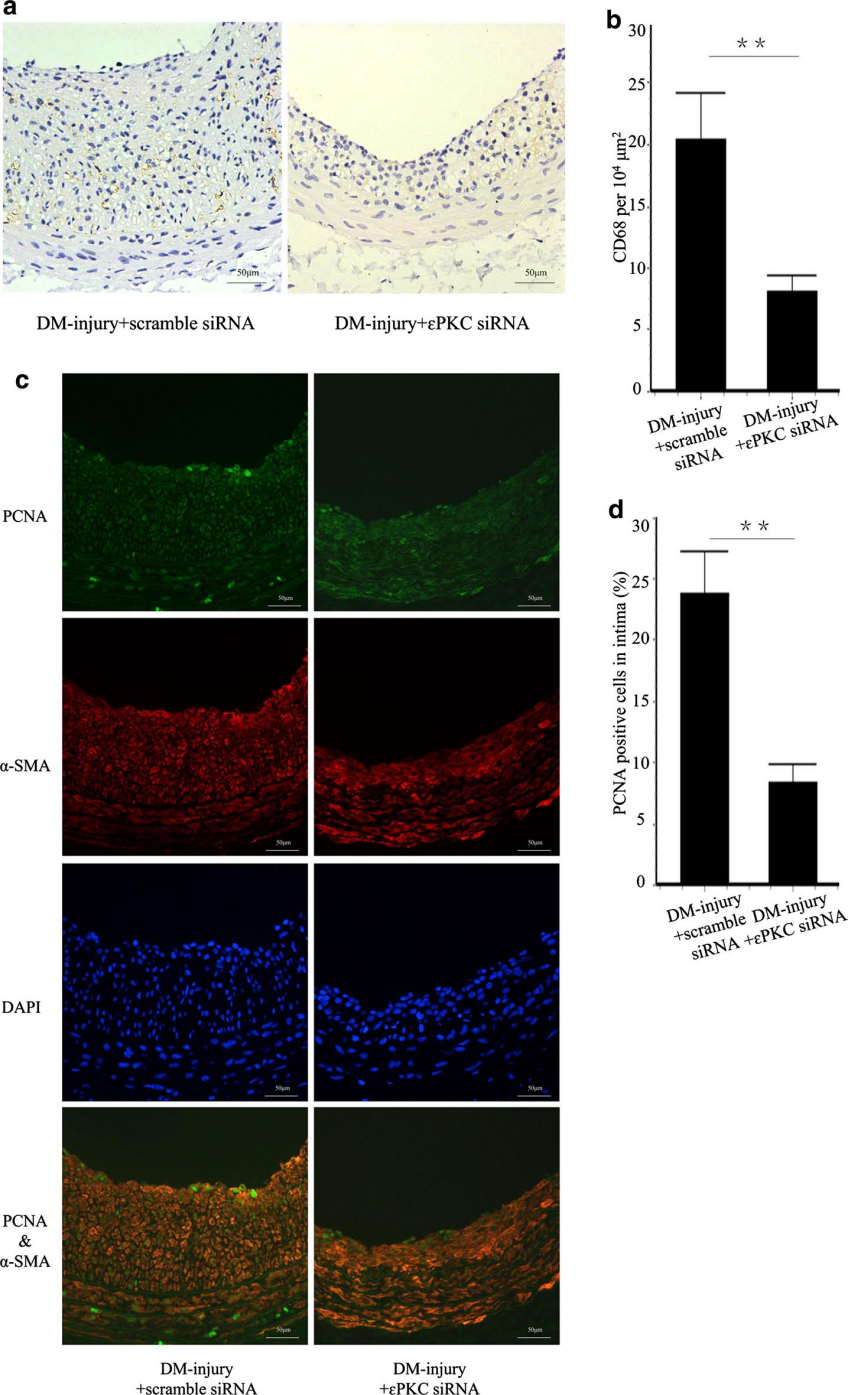
Effect of εPKC on inflammation and cell proliferation in balloon injured carotid arteries. **a** IHC stain of CD 68 was performed in carotid sections 14 days after balloon injury with or without εPKC siRNA delivery. **b** Quantitative analysis of CD 68-posotive cells per 10^4^ μm^2^. **c** IF staining of PCNA and α-SMA was performed in carotid sections 14 days after balloon injury with or without εPKC siRNA delivery. **d** Quantitative analysis of percentage of PCNA-positive cells in intima. Labeling of CD 68 and PCNA were significantly lower in εPKC siRNA delivered group than that in scramble siRNA delivered group. *Bars* represent mean ± SE. ** p < 0.01

Localized εPKC knockdown with targeted siRNA resulted in significant fewer CD 68-positive cells in intima than that in the scramble siRNA delivered group (8.14 ± 1.31 vs. 20.39 ± 3.75, p < 0.01) (Fig. 7a, b). IF staining of PCNA and α-SMA also show a decrease in PCNA-positive cells in intima in the εPKC siRNA delivered group (8.41 ± 1.56 % vs. 23.78 ± 3.45 %, p < 0.01) (Fig. 7c, d).

### εPKC regulates VSMCs proliferation and migration

**Fig. 8:**
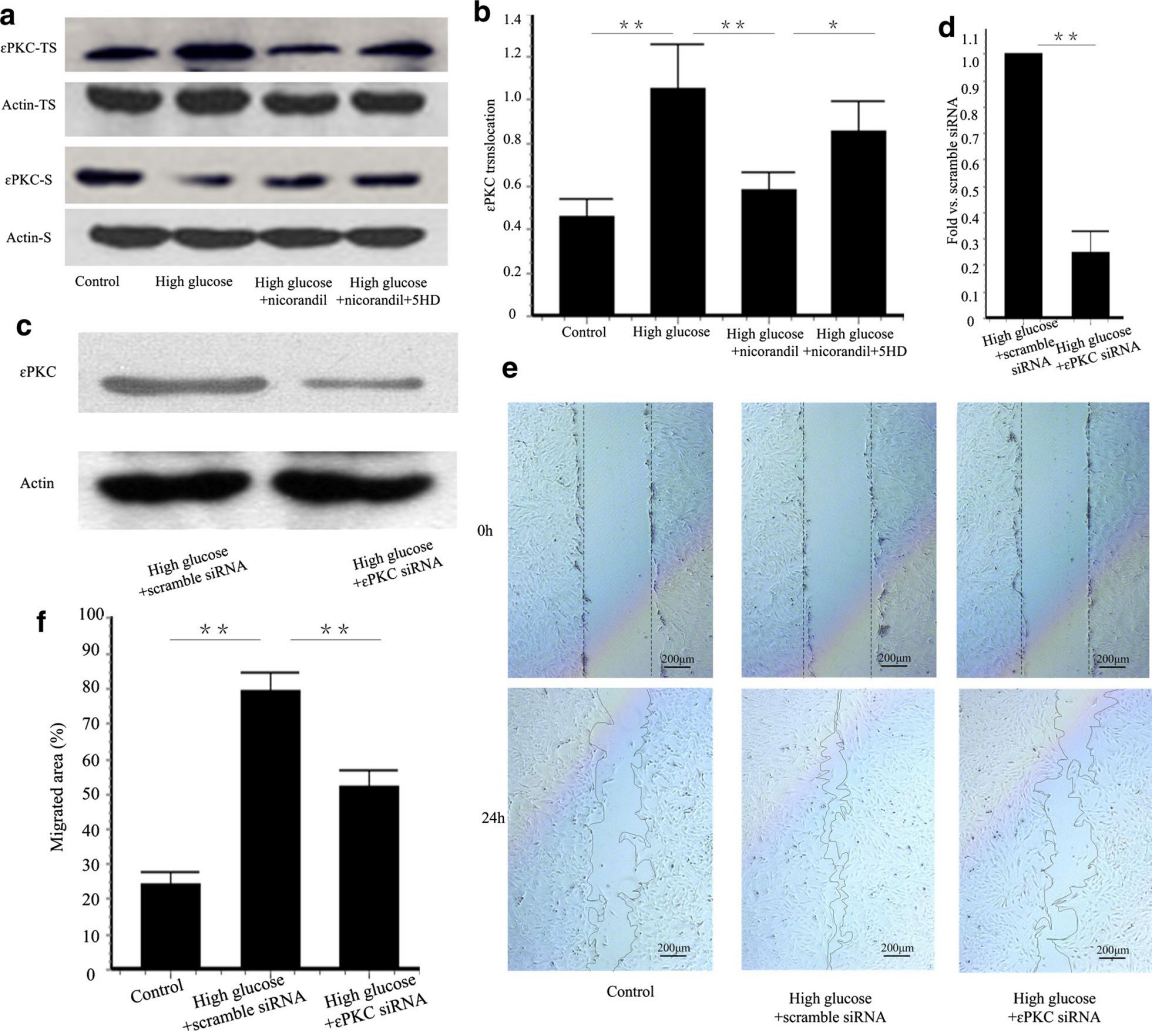
Effect of εPKC on VSMCs migration induced by high glucose. **a** Representative western blots for εPKC translocation in VSMCs with or without nicorandil or 5-HD. **b** Quantitative analysis of εPKC translocation. εPKC translocation was induced by high glucose. Nicorandil inhibited the increase of εPKC translocation by opening mitoKATP channel. **c** Representative western blots for εPKC protein in VSMCs with or without εPKC siRNA transfection. **d** Quantitative analysis of εPKC protein. εPKC protein in high-glucose-εPKC siRNA group was reduced to 24.94 ± 7.67 % of that in high-glucose-scramble siRNA group. **e** VSMCs migration was assessed by wound healing assay. **f** Quantitative analysis of percentage of migrated area in control group, high glucose + scramble siRNA group, and high glucose + εPKC siRNA group. εPKC siRNA knockdown prevented high glucose-induced cell migration. *Bars* represent mean ± SE. *p < 0.05, **p < 0.01

**Fig. 9:**
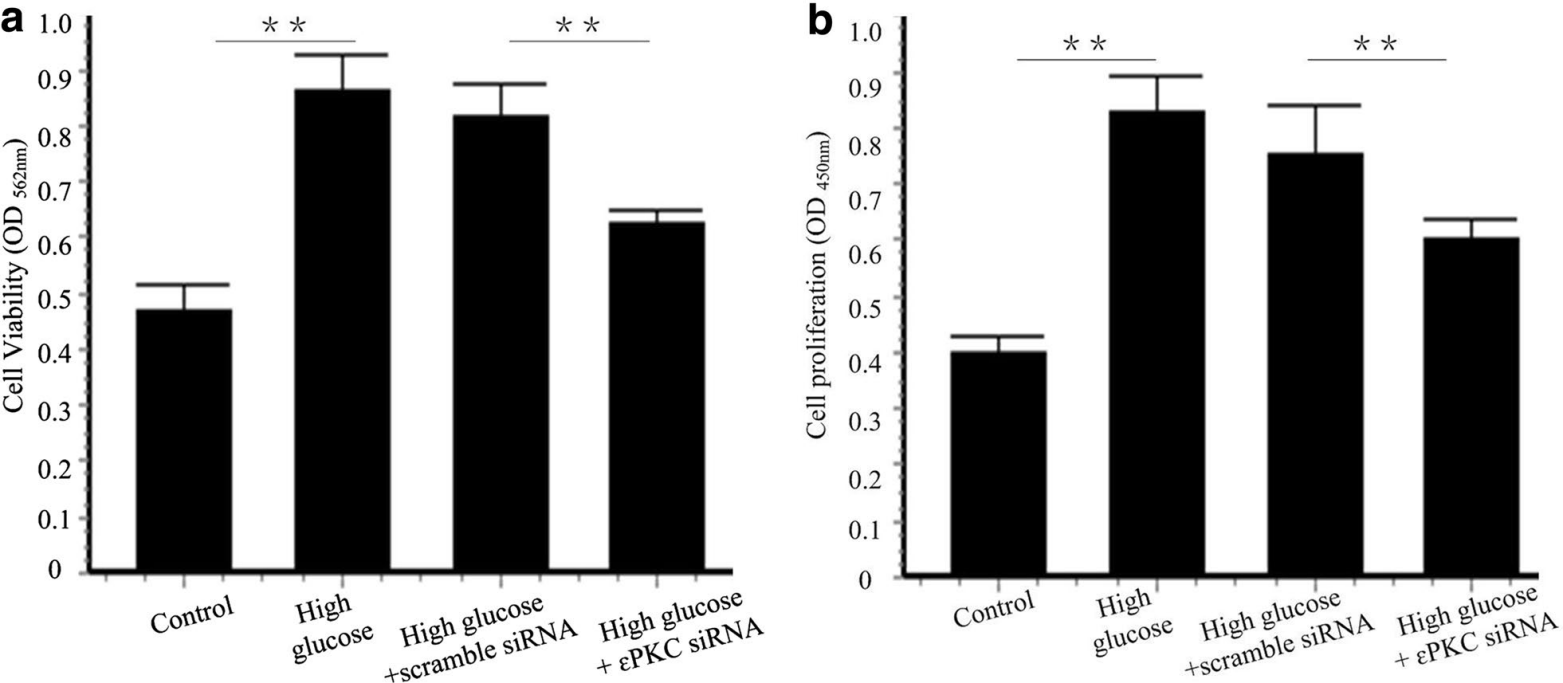
Effect of εPKC on VSMCs proliferation induced by high glucose. **a** Cell viability was assessed by MTT assay kit. **b** VSMCs proliferation was detected using BrdU proliferation assay kit. 24 h of high glucose induced VSMCs proliferation. εPKC knockdown suppressed the increase of cell viability and proliferation induced by high glucose. *Bars* represent mean ± SE. **p < 0.01

In previous experiments, high glucose induced εPKC translocation in primary cultured VSMCs [1]. In our present study, εPKC activation is partially blocked by nicorandil in vitro (Fig. 8a, b). Knockdown of εPKC was achieved 48 h after siRNA transfection. εPKC protein in high-glucose-εPKC siRNA group was reduced to 24.94 ± 7.67 % of that in high-glucose-scramble siRNA group (Fig. 8c, d). VSMCs migration stimulated by 24 h high glucose was significantly inhibited in the εPKC knockdown cells (79.42 ± 5.14 vs. 52.40 ± 4.66 %, p < 0.01) (Fig. 8e, f). Besides, cell proliferation and cell viability were also decreased in εPKC knockdown VSMCs, compared with the scramble siRNA transfected group (Fig. 9a, b).

## Discussion

In this study, we have demonstrated that nicorandil attenuates intimal hyperplasia and inflammation infiltration in arterial lesions in diabetic rats, and this is mediated by mitoK_ATP_ channel and εPKC pathway. Furthermore, our data indicates that nicorandil inhibits high glucose-induced VSMCs proliferation and migration. Our study may provide a better understanding about the effects of nicorandil on vascular injury in diabetic models.

Higher rate of restenosis is seen in diabetic patients compared to patients without diabetes after PCI [23]. Intimal hyperplasia arises from an initial injury to the artery. Diabetes enhances cellular proliferation, adhesion molecular expression and inflammatory cell infiltration after arterial injury in multiple animal models [24]. Diabetic rats respond to vascular injury with increased influx of inflammatory cells, enhanced proliferative activity and greater intima formation compared with non diabetic rats [25]. In vitro, high glucose promotes VSMCs migration and proliferation by increasing integrins, advanced glycation end products and glycoproteins [24]. Several anti-diabetic therapies have been found to inhibit intimal hyperplasia after balloon injury in preclinical model. Insulin detemir inhibits intimal hyperplasia after balloon catheter injury in Zucker Fatty rats [26]. Glucagon-like peptide-1(GLP-1)-based anti-diabetic agents, such as Dipeptidyl peptidase-4 inhibitor linagliptin [27], GLP-1 receptor agonist Exenatide [28], and GLP-1 analogue Liraglutide [29], have revealed their tissue-protective potentials. However, neutral protamine Hagedorn insulin does not inhibits intimal hyperplasia [26]. Potential therapies aimed at prevention of intimal hyperplasia are still needed. Nicorandil is a hybrid agent with ATP-sensitive potassium channel (K_ATP_) channel opener and nitrate properties. It is a widely used anti-angina agent and has shown vascular-protective effect in several clinical studies. Clinical study suggests that nicorandil reduces the rate of TVR [3]. Oral nicorandil also stabilizes coronary plaque in patients with stable angina pectoris [5]. A trend toward lower TLR was shown in diabetic patients undergoing PCI in a randomized control trial [4]. Coupled with the observation in these clinical studies, we observe that nicorandil significantly decreases intimal hyperplasia 14 days after balloon injury in diabetic rats. Nicorandil increases lumen area and decreases intima/media ratio and intima area after balloon injury in diabetic rats. Nicorandil also inhibits cell proliferation and macrophage infiltration in arterial lesions after 14 days of balloon injury. VSMCs account for 93.6 ± 1.72 % of the ultimate intimal proliferation. Previous experiments reported that nicorandil inhibited SMCs proliferation in rat pulmonary arterial hypertension model and attenuated LPS-induced inflammation [13]. Our data suggest that nicorandil reduces the rate of restenosis by inhibiting cell proliferation, reducing inflammation cell infiltration and attenuating the intimal hyperplasia after balloon injury in diabetic rats. In our preliminary experiment, we investigated the effect of nicorandil on intimal hyperplasia in non-DM rats. Nicorandil inhibits intimal hyperplasia after balloon injury in non-DM rats, but the differences of lumen area and I/M ratio between non-DM-injury group and non-DM-injury-nicorandil group are of no statistical significance (data shown in Additional file 1: Figure S1). The reason is that non-DM rats developed less intima than diabetic rats after balloon injury. With more predominant intima in DM rats, nicorandil exhibits inhibitive effect on intimal hyperplasia.

Previous studies on nicorandil in diabetic animals yields conflicting results. 15 mg/kg/day nicorandil has no effect on plasma glucose and body weight in STZ-induced diabetic rats [12]. 30 mg/kg/day nicorandil does not affect the blood glucose or body weight in STZ-injected mice [30]. 15 mg/kg/day nicorandil decreases the fast blood glucose and increases body weight, although this difference is not statistically significant [31]. When 0.003 % nicorandil-containing-diet is given 2 days after STZ injection, body weight loss and blood glucose level were significantly lower than those of STZ-injected rats. However, when nicorandil is given 3 or 4 days after STZ injection, no significant difference is observed between nicorandil treatment group and STZ-injected group [32]. In our preliminary study, nicorandil was given at the dose of 5 or 15 mg/kg/day from the 3rd day after STZ injection. 15 mg/kg/day nicorandil significantly inhibits intimal hyperplasia and is used in the later experiments. In the present study, 15 mg/kg/day nicorandil has no significant influence on body weight or glucose levels. The inhibition effect on intimal hyperplasia exhibited by nicorandil is not achieved through glycermic control.

Several lines of evidence suggest that high glucose per se promoted VSMCs proliferation and exaggerated intimal hyperplasia [1, 20]. In the present study, 24 h of high glucose treatment induces VSMCs proliferation and migration in the presence of 1 % serum. High glucose has been found to induce hyperpolarization of ΔΨm in vitro [20]. A recent study found that hyperpolarization of ΔΨm promotes VSMCs proliferation and intimal hyperplasia [8]. Nicorandil directly opens the mitoK_ATP_ channel and depolarize ΔΨm [9, 10]. To investigate the role of mitoK_ATP_ channel in the inhibition effect of nicorandil on intimal hyperplasia, 5-HD was used in vivo and in vitro. In vivo study reveals that 5-HD partially reverses the inhibition effect of nicorandil on intimal hyperplasia. Inflammation cell infiltration and proliferation inhibited by nicorandil was partially blocked by 5-HD. Inhibition effect of nicorandil on VSMCs proliferation and migration is also significantly blocked by pretreatment of 5-HD in vitro. Taken together, nicorandil inhibits intimal hyperplasia, inflammation, VSMCs proliferation and migration by opening mitoK_ATP_ channel.

PKC family consists of 11 related serine/threonine protein kinases. The importance of PKC signaling for VSMCs growth and restenosis has been shown previously. αPKC and εPKC, together with atypical PKCs, mediated cell proliferation and survival [33]. Sustained inhibition of εPKC inhibited intimal hyperplasia in vivo and prevented VSMCs proliferation and migration in vitro [7]. Chronic hyperglycemia leads to PKC activation and promotes VSMCs growth [1]. Nicorandil inhibits the activation of εPKC by opening mitoK_ATP_ channel in myocardial infarction rat model [11]. In the present study, we observe εPKC activation in balloon-injured arteries in the diabetic rats. Nicorandil inhibits this εPKC activation, and this inhibition effect was significantly reversed by 5-HD. The same εPKC activation trend is also observed in high glucose-stimulated VSMCs. In vivo εPKC knockdown is achieved by localized delivery of εPKC siRNA and identified by western blot. Knockdown of εPKC inhibits intimal hyperplasia in diabetic rats. When diabetic rats were treated with nicorandil and εPKC siRNA, the lumen area is larger than that of rats treated with nicorandil and scramble siRNA (data shown in Additional file 1: Figure S2). CD 68-positve macrophages and PCNA-positive cells in intima are also reduced by localized delivery of εPKC siRNA. In vitro εPKC knockdown is achieved by transfection of εPKC siRNA in VSMCs. εPKC siRNA transfection significantly reduces VSMCs migration area and proliferation. Thus, nicorandil inhibited εPKC activation by opening mitoK_ATP_ channel. This is the mechanism involved in the beneficial effect of nicorandil on intimal hyperplasia after balloon injury in diabetic models. Some previous studies reveal that opening of mitoK_ATP_ channel stimulates εPKC activation. In mouse insulinoma βTC-6 cells, NNC 55-0321, a novel potassium channel opener, promotes εPKC activation. However, NNC 55-0462, another novel potassium channel opener, does not promote εPKC activation [34]. In rat ventricular myocytes, diazoxide, a mitoK_ATP_ channel opener, induces εPKC translocation [35]. εPKC inhibitor cheleryhrine does not abolish infarct size-limiting effect of diazoxide in rabbit hearts, but it blocks the protective effect of diazoxide in rat myocardial infarction [19]. Nicorandil, diazoxide, NNC 55-3021 and NNC 55-0462 do not exhibit the same effect on εPKC activation in different animal models. The discrepancy may reflect species and organ differences in regulatory mechanisms of PKC and mitoK_ATP_ channel.

In other studies, nicorandil reduces the production of reactive oxygen species [13], prevents endothelial dysfunction [36], inhibits VACM-1 expression [31], attenuates cardiac sympathetic nerve injury [37], and interferences platelet aggregation [36]. These mechanisms may contribute to the inhibition of intimal hyperplasia; however, these are beyond the scope of this study. Future work is still needed to explore the detailed mechanisms of nicorandil on vascular injury, especially in diabetic models.

In summary, our data highlight the inhibitive effect of nicorandil on the intimal hyperplasia after balloon injury in diabetic rats and propose the underlying mechanism. Nicorandil opens mitoK_ATP_ channel, inhibits the activation of εPKC and prevents inflammation and cell proliferation in balloon-injured carotid arteries in diabetic rats. In vitro study reveals that nicorandil inhibits high glucose-stimulated VSMCs proliferation and migration by opening mitoK_ATP_ channel and inhibiting εPKC activation. No direct evidence of intimal hyperplasia inhibition has been observed in nicorandil-treated patients in clinical trials. Future clinical trials and basic studies should be performed to further reveal the protective effect of nicorandil on PCI-related complications.

## Conclusions

Our present study reveals for the first time that nicorandil attenuates carotid intimal hyperplasia after balloon injury in diabetic rats. Nicorandil inhibits cell proliferation and CD68-positive inflammatory cell infiltration in balloon-injured arteries of diabetic rats. The mechanism of the phenomenon involves opening of mitoK_ATP_ channel and inhibiting εPKC activation. In vitro study reveals that nicorandil inhibits high glucose-induced VSMCs proliferation and migration. These data suggest that nicorandil may have beneficial effect in diabetic patients undergoing PCI and reduce PCI-related complications.

## Availability of data and materials

The datasets supporting the conclusions of this article are included within the article.

## Additional file

10.1186/s12933-016-0377-6 Supplemental data.

## Abbreviations

STZ: streptozotocin
5-HD: 5-hydroxydecanoate
mitoK_ATP_ channel: mitochondrial ATP-sensitive potassium channel
VSMCs: vascular smooth muscle cells
PKC: protein kinase C
PCI: percutaneous coronary intervention
PTCA: percutaneous transluminal coronary angioplasty
DM: diabetes mellitus
TVR: target vessel revasculariztion
TLR: target lesion revascularization
ΔΨm: mitochondrial membrane potential
SD: Sprague-Dawley rats
H&E: hematoxylin and eosin
IEL: internal elastic lamina
EEL: external elastic lamina
IHC: immunohistochemistry
α-SMA: alpha smooth muscle actin
IF: immunofluorescent
MTT: modified 3-(4, 5-dimethyl-thiazol-2-yl)-2, 5-dyphenyltertrazolium bromide
CD68: cluster of differentiation 68
PCNA: proliferating cell nuclear antigen

## References

[1] Srivastava S, Ramana KV, Tammali R, Srivastava SK, Bhatnagar A (2006). Contribution of aldose reductase to diabetic hyperproliferation of vascular smooth muscle cells. Diabetes.

[2] Fröbert O, Lagerqvist B, Carlsson J, Lindbäck J, Stenestrand U, James SK (2009). Differences in restenosis rate with different drug-eluting stents in patients with and without diabetes mellitus. J Am Coll Cardiol.

[3] Kawai Y, Hisamatsu K, Matsubara H, Dan K, Akagi S, Miyaji K (2009). Intravenous administration of nicorandil immediately before percutaneous coronary intervention can prevent slow coronary flow phenomenon. Eur Heart J.

[4] Shehata M (2014). Cardioprotective effects of oral nicorandil use in diabetic patients undergoing elective percutaneous coronary intervention. J Interv Cardiol..

[5] Izumiya Y, Kojima S, Kojima S, Araki S, Usuku H, Matsubara J (2011). Long-term use of oral nicorandil stabilizes coronary plaque in patients with stable angina pectoris. Atherosclerosis.

[6] Group TIS (2002). Effect of nicorandil on coronary events in patients with stable angina: the impact of nicorandil in angina (iona) randomised trial. Lancet.

[7] Deuse T, Koyanagi T, Erben RG, Hua X, Velden J, Ikeno F (2010). Sustained inhibition of protein kinase c inhibits vascular restenosis after balloon injury and stenting. Circulation.

[8] Deuse T, Hua X, Wang D, Maegdefessel L, Heeren J, Scheja L (2014). Dichloroacetate prevents restenosis in preclinical animal models of vessel injury. Nature.

[9] Dymkowska D, Drabarek B, Jakubczyk J, Wojciechowska S, Zabłocki K (2014). Potassium channel openers prevent palmitate-induced insulin resistance in C2C12 myotubes. Arch Biochem Biophys.

[10] Ishida H, Higashijima N, Hirota Y, Genka C, Nakazawa H, Nakaya H (2004). Nicorandil attenuates the mitochondrial Ca2+ overload with accompanying depolarization of the mitochondrial membrane in the heart. Naunyn Schmiedebergs Arch Pharmacol.

[11] Lee T-M, Lin C-C, Lien H-Y, Chen C-C (2012). KATP channel agonists preserve connexin43 protein in infarcted rats by a protein kinase C-dependent pathway. J Cell Mol Med.

[12] Mano T, Shinohara R, Nagasakacorrespondence A, Nakagawa H, Uchimura K, Hayashi R (2000). Scavenging effect of nicorandil on free radicals and lipid peroxide in streptozotocin-induced diabetic rats. Metabolism.

[13] Lee F-Y, Lu H-I, Zhen Y-Y, Leu S, Chen Y-L, Tsai T-H (2013). Benefit of combined therapy with nicorandil and colchicine in preventing monocrotaline-induced rat pulmonary arterial hypertension. Eur J Pharm Sci.

[14] Cheng Y, Makarova N, Tsukahara R, Guo H, Shuyu E, Farrar P (2009). Lysophosphatidic acid-induced arterial wall remodeling: requirement of PPAR γ but not LPA1 or LPA2 GPCR. Cell Signal.

[15] Qu Y, Shi X, Zhang H, Sun W, Han S, Yu C (2009). VCAM-1 siRNA reduces neointimal formation after surgical mechanical injury of the rat carotid artery. J Vasc Surg.

[16] Smolock EM, Korshunov VA, Glazko G, Qiu X, Gerloff J, Berk BC (2012). Ribosomal protein L17, RpL17, is an inhibitor of vascular smooth muscle growth and carotid intima formation. Circulation.

[17] Redmond EM, Liu W, Hamm K, Hatch E, Cahill PA, Morrow D (2014). Perivascular delivery of notch 1 siRNA inhibits injury-induced arterial remodeling. Plos ONE..

[18] Hlawaty H, Suffee N, Sutton A, Oudar O, Haddad O, Ollivier V (2011). Low molecular weight fucoidan prevents intimal hyperplasia in rat injured thoracic aorta through the modulation of matrix metalloproteinase-2 expression. Biochem Pharmacol.

[19] Tsuchida A, Miura T, Tanno M, Sakamoto J, Miki T, Kuno A (2002). Infarct size limitation by nicorandil: roles of mitochondrial KATP channels, sarcolemmal KATP channels, and protein kinase C. J Am Coll Cardiol.

[20] Matsuoka T, Wada J, Hashimoto I, Zhang Y, Eguchi J, Ogawa N (2005). Gene delivery of Tim44 reduces mitochondrial superoxide production and ameliorates neointimal proliferation of injured carotid artery in diabetic rats. Diabetes.

[21] Lee T-H, Sottile J, Chiang H-Y (2015). Collagen inhibitory peptide R1R2 mediates vascular remodeling by decreasing inflammation and smooth muscle cell activation. PLoS ONE.

[22] Shyu K-G, Wang B-W, Kuan P, Chang H (2008). RNA interference for discoidin domain receptor 2 attenuates neointimal formation in balloon injured rat carotid artery. Arterioscler Thromb Vasc Biol.

[23] Fröbert O, Lagerqvist B, Carlsson J, Lindbäck J, Stenestrand U, James SK (2009). Differences in restenosis rate with different drug-eluting stents in patients with and without diabetes mellitus : a report from the SCAAR (Swedish Angiography and Angioplasty Registry). J Am Coll Cardiol.

[24] Fu Y, Duru EA, Davies MG (2014). Effect of metabolic syndrom and the response to arterial injury. J Surg Res.

[25] Barbato JE, Zuckerbraun BS, Overhaus M, Raman KG, Tzeng E (2005). Nitric oxide modulates vascular inflammation and intimal hyperplasia in insulin resistance and the metabolic syndrome. Am J Physiol-Heart C..

[26] Murthy SN, Pankey EA, Banka AA, Badejo AM, Wekerle R, Vilija V (2012). Effects of insulin detemir on balloon catheter injured carotid artery in Zucker fatty rats. J Diabetes Complicat.

[27] Terawaki Y, Nomiyama T, Kawanami T, Hamaguchi Y, Takahashi H, Tanaka T (2014). Dipeptidyl peptidase-4 inhibitor linagliptin attenuates neointima formation after vascular injury. Cardiovas Diabetol.

[28] Murthy SN, Hilaire R-CS, Casey DB, Badejo AM, McGee J, McNamara DB (2010). The synthetic GLP-1 receptor agonist, exenatide, reduces intimal hyperplasia in insulin resistant rats. Diabetes Vasc Dis Res..

[29] Shi L, Ji Y, Jiang X, Zhou L, Xu Y, Li Y (2015). Liraglutide attenuates high glucose-induced abnormal cell migration, proliferation, and apoptosis of vascular smooth muscle cells by activating the GLP-1 receptor, and inhibiting ERK1/2 and PI3 K/Akt signaling pathways. Cardiovas Diabetol.

[30] Tanabe K, Lanaspa MA, Kitagawa W, Rivard CJ, Miyazaki M, Klawitter J (2012). Nicorandil as a novel therapy for advanced diabetic nephropathy in the eNOS-deficient mouse. Am J Physiol-Renal.

[31] Liu L, Liu Y, Qi B, Wu Q, Li Y, Wang Z (2014). Nicorandil attenuates endothelial VCAM-1 expression via thioredoxin production in diabetic rats induced by streptozotocin. Mol Med Rep.

[32] Kasono K, Yasu T, Kakehashi A, Kinoshita N, Tamemoto H, Namai K (2004). Nicorandil improves diabetes and rat islet b-cell damage induced by streptozotocin in vivo and in vitro. Eur J Endocrinol.

[33] Churchill EN, Mochly-Rosen D (2007). The roles of PKC delta and epsilon isoenzymes in the regulation of myocardial ischaemia/reperfusion injury. Biochem Soc Trans.

[34] Sandler S, Andersson AK, Larsson J, Makeeva N, Olsen T, Arkhammar POG (2008). Possible role of an ischemic preconditioning-like response mechanism in KATP channel opener-mediated protection against streptozotocin-induced suppression of rat pancreatic islet function. Biochem Pharmacol.

[35] Li H, Yang T, Long Z, Cheng J (2014). Effect of mitochondrial ATP-sensitive potassium channel opening on the translocation of protein kinase C epsilon in adult rat ventricular myocytes. Genet Mol Res.

[36] Aizawa K, Takahari Y, Higashijima N, Serizawa K, Yogo K, Ishizuka N (2015). Nicorandil prevents sirolimus-induced production of reactive oxygen species, endothelial dysfunction and thrombus formation. J Pharmacol Sci..

[37] Miura T, Kawamura S, Tatsuno H, Ikeda Y, Mikami S, Iwamoto H (2001). Ischemic preconditioning attenuates cardiac sympathetic nerve injury via atp-sensitive potassium channels during myocardial ischemia. Circulation.

