# Supplementary material for: Nicorandil attenuates carotid intimal hyperplasia after balloon catheter injury in diabetic rats
Source: Cardiovasc Diabetol. 2016 Apr 8;15:62. doi: 10.1186/s12933-016-0377-6 (PMC4826484; doi:10.1186/s12933-016-0377-6)
Supplement: Supplementary file 1 — 10.1186/s12933-016-0377-6 Supplemental data. [file 12933_2016_377_MOESM1_ESM.docx]

**Nicorandil attenuates carotid intimal hyperplasia after balloon catheter injury in diabetic rats**

Figure S1a


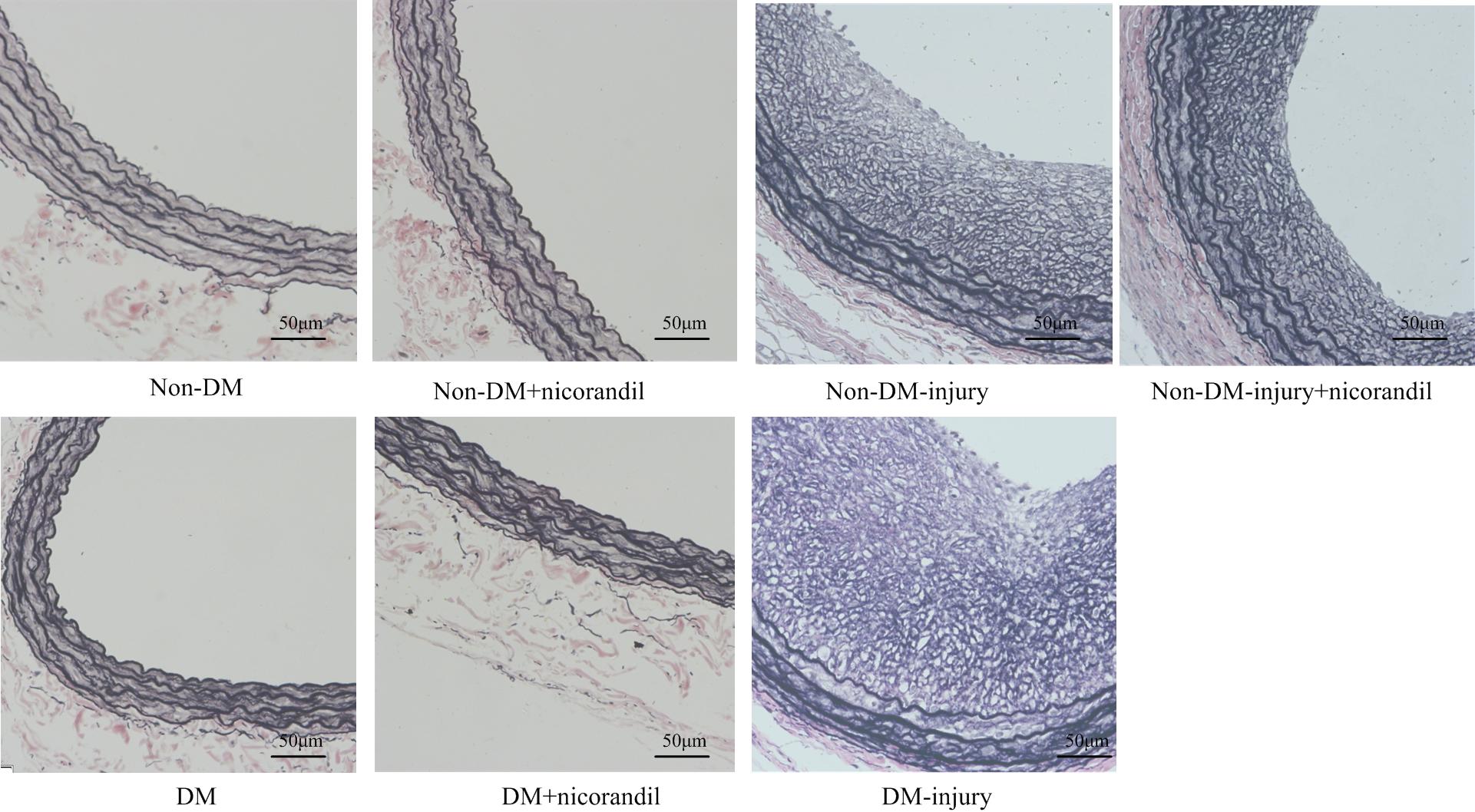


Figure S1b


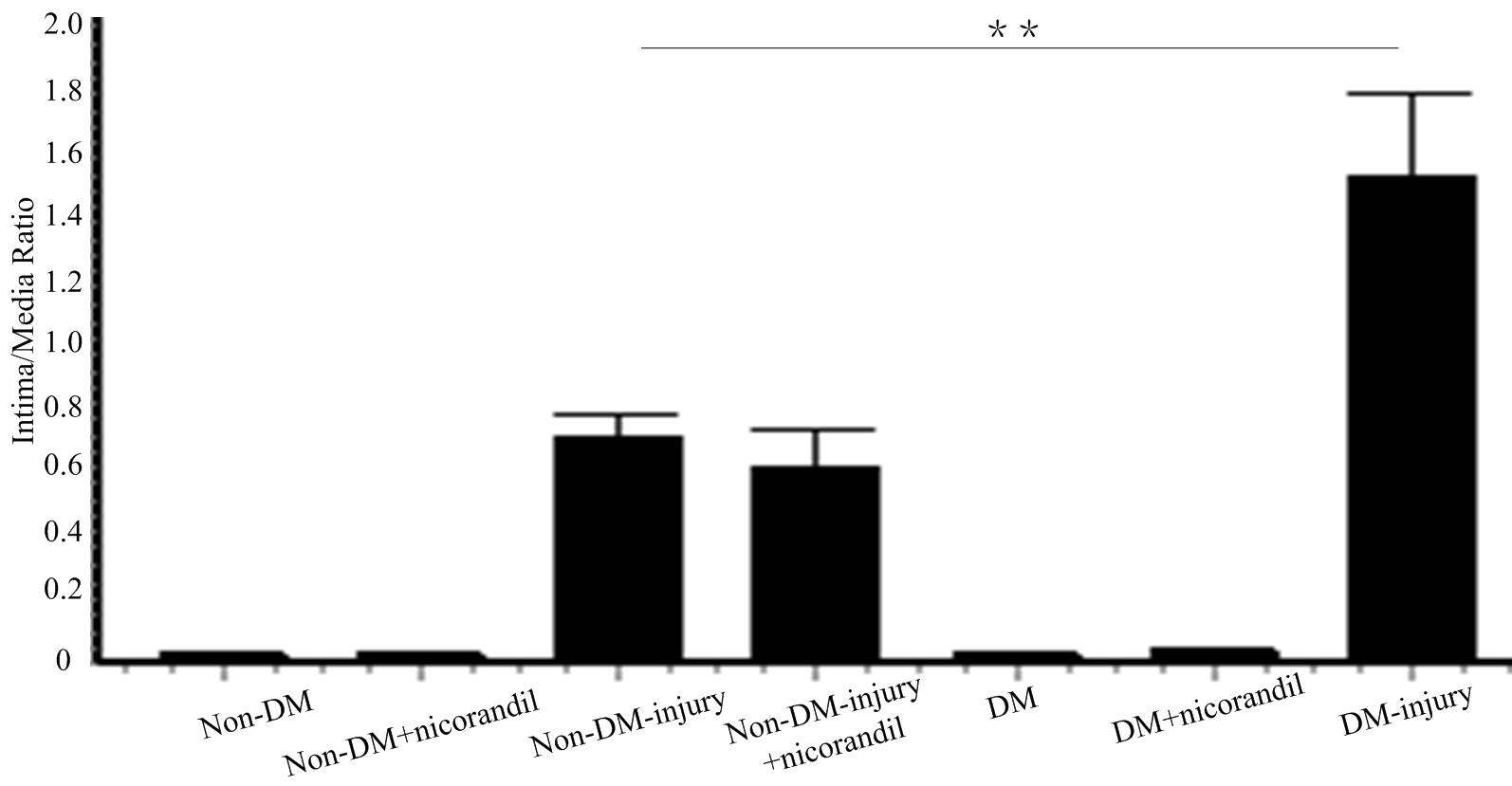


Figure S1c


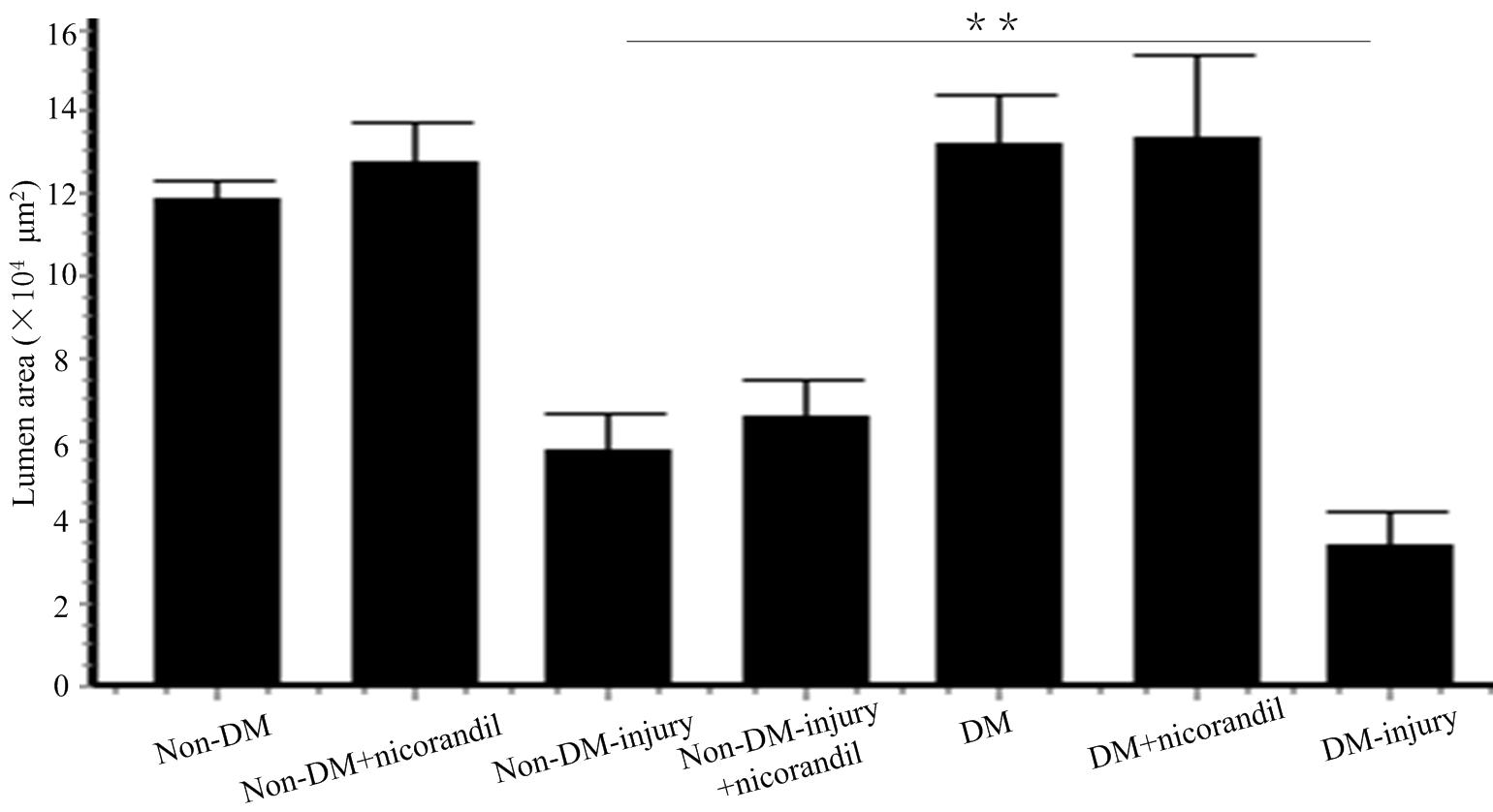


Figure S1. Intimal hyperplasia of carotid arteries in different groups. a: Cross sections of carotid arteries from diabetic rats 14 days after balloon injury. Sections were stained with Elastica van Gieson. b: Quantitative analysis of intima to media area ratio. There were no intimal hyperplasia in non-DM, non-DM+nicorandil, DM, and DN-nicorandil group. The difference of Intima/Media ratio between non-DM-injury group and non-DM-injury+nicorandil is of no statistical significance. The difference of I/M ratio between non-DM-injury group and DM-injury group is of statistical significance. c: Quantitative analysis of lumen area. The difference of lumen area between non-DM-injury group and non-DM-injury+nicorandil group is of no statistical significance. The difference of lumen area between non-DM-injury group and DM-injury group is of statistical significance. Bars represent means±SE. **p<0.01.

Figure S2a Figure S2b Figure S2c


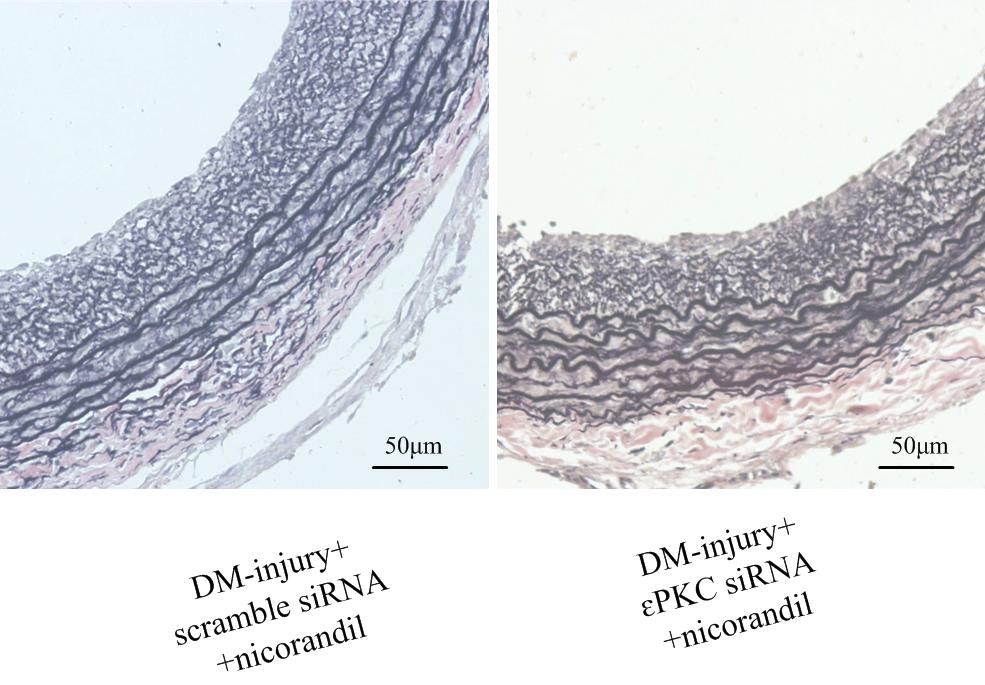

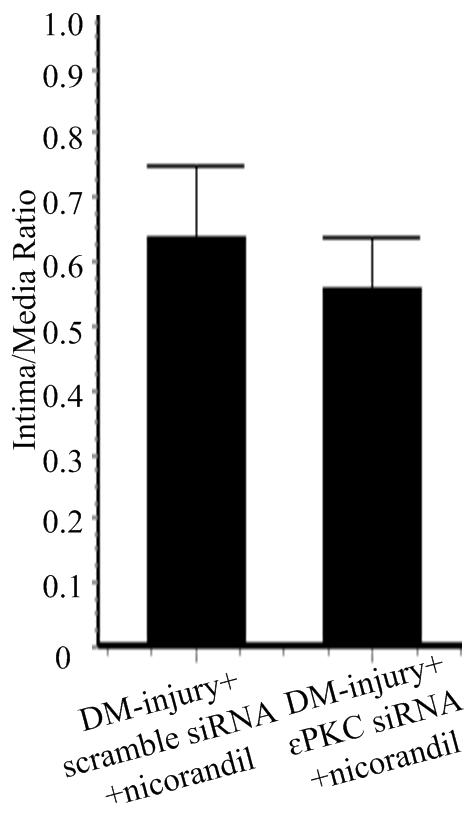

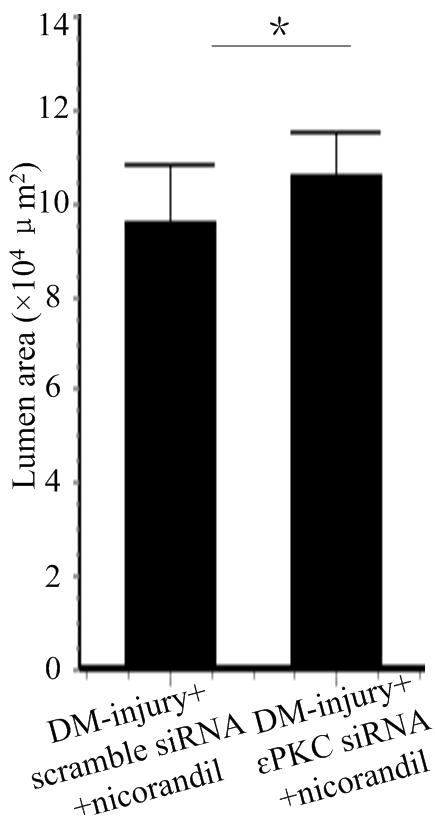


Figure S2. Cross sections of carotid arteries in DM-injury+scramble siRNA+nicorandil group and DM-injury+εPKC siRNA+nicorandil group. a: Cross sections of carotid arteries from diabetic rats 14 days after balloon injury. Sections were stained with Elastica van Gieson. b: Quantitative analysis of intima area to media area ratio. I/M ratio in DM-injury+εPKC siRNA+nicorandil group is lower than that in DM-injury+scramble siRNA+nicorandil group. However, the difference is of no statistical significant. c: Quantitative analysis of lumen area. Lumen area in DM-injury+εPKC siRNA+nicorandil group is larger than that in DM-injury+scramble siRNA+nicorandil group. The difference is of statistical significant. Bars represent means±SE. *p<0.05.
